# Remarks on the Juribali, or Euribali, (so Called by the Natives,) a Febrifuge Bark Tree of Pomeroon

**Published:** 1833-01

**Authors:** John Hancock

**Affiliations:** Fellow of this Society, Honorary Member of the Society of Arts of Scotland, &c.


TRANSACTIONS OF THE MEDICO-BOTANICAL SOCIETY OF LONDON.

*Published under the Direction of the President and Council.*

### THE JURIBALI.

**Remarks on the Juribali, or Euribali,** *(so called by the Natives,) a Febrifuge Bark Tree of Pomeroon.* By John Hancock, m.d., Fellow of this Society, Honorary Member of the Society of Arts of Scotland, &c.

This tree is found in the forests not far distant from the coast. It is small, seldom exceeding thirty feet in height, and eight or ten inches in diameter at the base. It belongs to the eighth class and first order of the sexual system of Linnæus, and to the natural family of Meliaceæ of Jussieu. The calyx is very small, of one leaf, entire. The corolla consists of four petals, lance-ovate, white, spreading. The nectarium is a monophyllous, bell-shaped tube, eight-toothed, bloated or inflate, bearing the stamina in its clefts or notches: this part is described by several authors as the filaments united. The stamina are without filaments. The anthers are eight in number, ovate, erect, seated upon the mouth of the nectarium. The germ is obtusely conic and pubescent. The style is very short, bearing capitate, or rather coronate, stigmata. The pericarpium is a capsule, ovate, one-celled, trivalved, the valves bearing rudiments of septa at their extremities: it contains a single seed, which is roundish, black, crowned with a trifid wing, arillate on one side only; it is veined, and resembles the nutmeg in shape, but is only half its size, with a fleshy albumen and foliaceous cotyledons. The flowers are numerous, on long, lax, divaricate panicles.

Nature has distinguished this tree in a very remarkable manner; for it may be truly said to bear two distinct kinds of leaves, the stipules being, at certain seasons, so developed as to be not unfrequently confounded with the common leaves of the tree, but are distinguished by their shape and position. They are placed in pairs, and scattered along the branches; ear-shaped or rounded and varied, obtuse and petiolate.

The common or proper leaves are alternate, oblong, pointed; they are scattered, without much order, on the branches; the petioles are short, compressed, and channelled. The bark is rough and gray externally, and, on peeling it from the tree, the epidermis scales off, and leaves the true bark of a smooth red surface. Its odour is peculiar, somewhat like that of tea-leaves. The wood of the trunk is dense and whitish; that of the branches somewhat coloured, and traversed by a pith in the centre. The seed, crowned with a foliaceous appendage, corresponds with that of the first tribe, Melieæ, of De Candolle; in other respects, to his second tribe, Trichilieæ.*

In respect to the calyx (being quite entire), it seems unique, as all the others of the order are divided or dentate. The structure of the flower, in all other respects, is strictly conformable to the order Meliaceæ; whilst, in the fruit, (a single-seeded capsule,) it agrees with very few of them: in one species only, Trichilia moschata, we observe it noted *Capsulis submonospermis.*

The most remarkable disparity, however, seems to be in the presence of stipulæ, which have hitherto in no instance been observed in this order.

The Juribali, therefore, will be found, I presume, to constitute a distinct genus from any yet described: it so appears,

* The learned author has this remark on the order Meliaceæ: "Ordo non satis definitus et forsan typos plurimos diversos colligius sed ob descriptiones plurium generum maneas, in statu scientiæ præsenti extricatu difficillimus et botanicis heritis commendandus." Pars i. p. 619.

at least, by comparing it with the forty-fourth order in De Candolle's Prodromus. The admirable arrangement, conciseness, and precison of this work enables us, at one glance almost, to observe the actual state of the science, so far as it goes, and, when completed, it will furnish an invaluable treasure to the botanist.

The bark of the Juribali gives a deep and lively red colour to water and spirit, in both of which its virtues are very soluble. It is a very potent bitter and astringent; in these qualities much exceeding the Peruvian bark, and will often be found to succeed after the latter has failed to remove an intermittent. I have commonly employed it in about half the quantity I should do for a corresponding dose of the Peruvian bark, to which, in fevers of a malignant and typhoid nature, it appears to be far superior. Notwithstanding its astringency, it does not, like Peruvian bark, constipate the bowels or affect the head, but generally opens the pores of the skin and promotes diaphoresis. To render it still more effectual, it should be taken warm.

More than one fourth the weight of this bark is soluble in water, whilst, according to Fabroni's experiments, the cinchona yields but about one sixth or one eighth. The active principle appears to be readily soluble in aqueous menstrua, and is therefore taken with much more facility than an insoluble woody mass, which passes the throat with difficulty and disgust, and often lies a heavy indigestible load on the stomach. Such considerations may one day appear of more importance than at present, when the prevailing infatuations respecting quinine and the new alkaloids shall have subsided, and given place to the exercise of sober reason and the examination of new doctrines by careful experiment.

I shall here notice the results of a few chemical experiments made on this bark, although I must confess I consider their action to be of very little consequence in elucidating the medicinal powers of any vegetable remedy. Gelatin forms with the infusion a precipitate of a reddish brown colour. Emetic tartar, nitrates of silver and mercury, acetates of lead and of alumina, all throw down precipitates of a light yellow colour; sulphate of copper affords a gray, and sulphate of iron a greenish blue precipitate. The carbonates of potash and soda render the infusion red brown, but form no precipitate. Lime water first renders the infusion green, then deep red, and throws down a copious precipitate of the same colour.

These experiments were made for the sake of comparison, consecutively with others, on infusions of cinchona of more marked sensible qualities, but which I could not refer with any certainty to their species. The results were in some cases similar, in others widely different in respect to the action of reagents; i.e. on the infusion of the Juribali and the cinchonas.

The recent decoction or infusion is of a red colour, but remains turbid for some days. After infusing it for two or three weeks, it gradually assumes a deep red tinge, more transparent, having deposited a flocculent sediment. In this state it gives a durable red colour to stuffs, and precipitates the infusion of galls, which the recent infusion does not.

It hence seems to be probable that, by a combination, or through some slight acidity, the infusion possesses the power of dissolving an alkaline principle, perhaps cinchonine, which is not taken up by pure water, or at least is not indicated in the recent infusion. It might be interesting to ascertain if the sulphuric or muriatic acids would evolve an alkaloid similar to those which are found in certain species of the genus cinchona.

The bark contains a resinoid extractive, which is soluble in boiling water, but not in cold; the decoction, therefore, becomes turbid on cooling, and gradually deposits a red powder. This deposit is soluble in alcohol, and appears to be a simple resin, and not the active principle of the bark; it is insipid when washed in cold water. From this, and some other experiments, I concluded that *cold* water took up the a*ctive* parts as well as hot.

This bark not only cures intermittent fevers, but remittents, also those of a typhoid malignant kind, and those destructive fevers in which the cinchona often does more harm than good. In some measure it emulates rhubarb, being cordial and purgative according to the dose; it is also a powerful diaphoretic, especially if taken warm, by which its value is certainly much enhanced as a febrifuge. I have used it in agues and in the malignant remittent fevers of the tropics, very freely, with the most decisive success, always in the form of infusion, commencing at any time or stage of the fever that may be present. By infusing an ounce of the bark in a quart of hot water, and giving a glassful once in two or three hours, I think it bids fair to be a useful remedy in smallpox and measles.

In a few instances the pulse seemed to be accelerated after its use, but was generally rendered slower and fuller; but I never ascertained the conditions of the patients under which these different effects took place, to my own satisfaction. Before quitting the present subject, I beg leave to allude to an opinion which has long prevailed in my mind, and which may be a novel one, or may not; but I have never heard or seen it adverted to, and, if correct, it may be worthy the candid consideration of the members of this Society and the profession at large.

We must all admit the great value and important advantages derived from the Peruvian bark in the practice of medicine. It is chiefly in fevers that its uses are to be regarded as paramount: but let us consider how far it is entitled to such unlimited encomiums as a febrifuge. It is certainly excellent as a tonic, and, as such, is applicable in the treatment of very many disorders. It affords one of the most efficient means of suspending the returns of the *common* intermittents. I say common, because, in the very malignant and bilious intermittent fevers of warm countries at least, often met with, it does more harm than good; and in the ardent, typhoid, and remittent fevers, and where most danger lies, we find its uses to be the most equivocal, and not unfrequently to produce a fatal metastasis on the brain: and in such fevers, those of the most dangerous tendency, it is rarely prescribed till the fever has subsided, when the skin has become moist, the tongue cleaning, sediment in the urine, &c., before the main remedy can be exhibited; and thus the time must be frittered away in *expectancy*, whilst the disease is making its inroads, until it has worn itself out, and the principle of life perhaps along with it.* When debility is the chief symptom prevailing, and when, in most cases, the danger is actually over, the bark is thrown in, and gets the merit of the cure! Under this view, therefore, it seems to me that its uses are not so strictly what its title of *febrifuge* imports. It is not so much *to drive away* the fever as to prevent its recurrence, when nine times in ten *dangerous* remittent fevers will not recur after once coming to a crisis. A real and genuine febrifuge, I should conceive, is that which not only braces the nerves as a preventive, but which is capable of *driving away* or taking off the febrile paroxysm. Such is the true meaning I should attach to a febrifuge or an anti-febrile remedy, and as such I conceive the remedy here recommended to be. But I shall leave the subject to the examination of better qualified judges.

This is, as before mentioned, but a small tree; there is another which grows very large, often confounded under the same name by the Arowaks: it is the Icica Altissima of Aublet. The remarkable large stipula, however, distinguishes

* Our profession has given too much reason for the satire of Voltaire, when he remarks, that "Nature cures diseases, and the physician assumes the credit."

the right kind most readily from every other tree which might otherwise resemble it; the scaly cuticle is also a good mark of distinction.

My experience is chiefly confined to its use in fever, but it may doubtless be regarded as a general tonic, and applicable, perhaps, in most cases as a substitute for the cinchona; externally it is found to be a very useful application to foul and ill-conditioned ulcers, either in powder or decoction.

There is another tree of the inland parts, called Caramata and *Arumari* by the natives, which affords likewise a very valuable remedy, a very bitter bark, which, from many trials I have made in those cases, appears to be equally safe and efficacious in those dangerous typhoid and remittent fevers in which the cinchona is either useless or pernicious, especially when exhibited during the febrile excitement. Being partial to the combination of similar remedies, I have in a few instances, when both happened to be at hand, infused the two barks (Juribali and Caramata) together, half an ounce of each, grossly powdered, to a quart of boiling water, giving the patient a wineglassful of the infusion, kept warm before the fire, once in two to four or six hours, according to the urgency of the case, and it has appeared to me to operate in this way with uncommon efficacy: but no one person singly is fit to decide upon the positive or comparative merits of a new remedy; and I shall, with great pleasure, submit these two medicinal barks, for further proofs and experiments, to the learned members of this Society, together with some imperfect botanical specimens of the trees from which they are procured; and, being soon to return to British Guiana, shall not fail to forward to the Society sufficient supplies* for making the requisite trials of their virtues, being fully convinced that no institution can afford such advantages as the Medico-Botanical Society for proving and fully investigating all the details which are requisite for the complete development of the powers of new remedies, and for deciding on their real or imaginary virtues.

It is only necessary to look at the assemblage at one of the general meetings, or at a list of the members of this Society, to be convinced of the truth of the remark which I have just made. We there behold a phalanx of talent, of the first order of botanists, chemists, physiologists, and the votaries of general science, combining their powers for the purpose of

* I have been extremely disappointed of these, and numerous other interesting articles, which were promised to be forwarded from the same quarter, during my abode in England.

forwarding objects of the most vital importance to our species. To all these facilities are added the great paramount advantage, that many of the learned members, physicians and general practitioners, besides an extensive private practice, are connected with hospitals where numerous patients are under immediate inspection, affording altogether the most adequate means of instantly submitting any remedy to the test of clinical experiment.

It might perhaps be considered invidious to designate any of the eminent members in support of my assertions, I may however observe, that I could mention very many names calculated to bestow celebrity, and to strengthen the great and worthy cause in which this Society is engaged. It is sufficient here to mention that the distinguished presidents of the College of Physicians, and of the College of Surgeons, are amongst the number, as well as many others, scarcely less eminent, and, without disparagement, we may say of equal talents. If the old adage, borrowed from the Romans, be true "in union is strength," we can scarcely wish for a better assurance of prosperity, and especially whilst the Society has the further guarantee of success, from the exertions of so zealous, learned, and distinguished a nobleman as presides over it, and at present guides the helm; who, without professing the Æsculapian art, is yet deeply read in it, and especially attached to the much-neglected, but most important, department of medical botany, and, although not a medical practitioner, yet a practiser of charity, and, I may add, a real friend to the poor and a patriot of his country.

Some may imagine that this is irrelevant to our subject, but I entertain a different opinion. The objects we should hold ourselves pledged to maintain are, and I hope ever will be, the charitable purposes of aiding the poor, and of turning the bounties of nature to the relief of human suffering.

---

